# Contacts with limited interpenetration

**DOI:** 10.1016/j.mex.2022.101688

**Published:** 2022-04-06

**Authors:** Jiří Jarušek

**Keywords:** Limited interpenetration, Quasistatic contact problems, Coulomb friction, Approximate problems, Existence of solutions, Relation to the Signorini contact

## Abstract

The aim of this paper is to acquaint a wider public of applied mathematicians, numerical analysts and engineers with the model of contact with limited interpenetration as a suitable framework for computation of practical problems. It is mostly based on the newly published Ref. [5]. The model is physically well based on the microscopic structure of a standard material of a body being in an actual or potential contact with a rigid foundation. Such microscopic phenomena are macroscopically interpreted as a certain but strictly limited surface interpenetration of both objects. The essence of this interpenetration is depicted in the graphical abstract. After a brief description of its motivation and the method itself, a comparison with the other contact models available together with the detailed description of the graphical abstract is presented. Furthermore, the application of the method to a quasistatic frictional boundary contact is described. Moreover, a brief description of the methods used in the proof of the existence of solutions of such contact problems is provided. If the depth of the interpenetration tends to zero, then there is some sequence of solutions of such problems and some solution to the corresponding Signorini contact problem such that it is the limit of the sequence. Requirements for the use of the presented model in solving practical problems as well as its other aspects are briefly discussed. Summing up:

• the presented and other results published (Refs. [1], [2], [3], [4]) create a reliable basis of the numerical analysis of the problems;
• the method is ready to be used in solving a wide class of contact problems arising in technical practice.

## Graphical abstract

**Figure:**
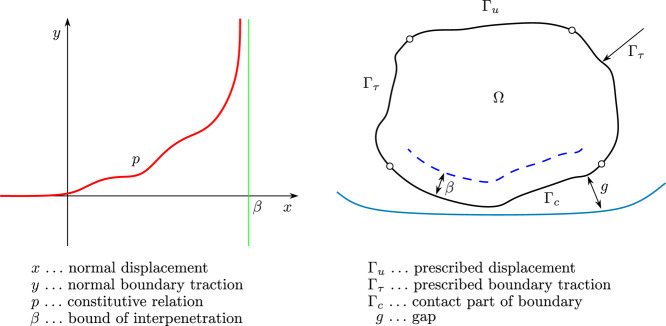

Specifications table

**Table:** 

Subject Area:	Engineering
More specific subject area:	*Unilateral contact problems in mechanics*
Method name:	*Contact with limited interpenetration.*
Name and reference of original method:	*It was for the first time treated in the paper**[1]**, cf. References.*
Resource availability:	*J. Jarušek. On the existence of solutions to quasistatic rational contact prob-lems with limited interpenetration. Nonlin. Anal. Real World Appl. 65 (2022).*

## Method details

In computation of contact problems mostly describing the contact of a deformable body with a foundation or a contact of two deformable bodies two frameworks from continuum mechanics have been considered up-to-now:

1. the Signorini model where the impenetrability of Mass is assumed. It started the research in contact problems, cf. Refs [7] and [8],
2. the so-called normal compliance model allowing in fact an unlimited interpenetrability of both objects.

While the first approach may be a bit idealized, the second one does not seem to respect the mechanical reality.

Looking at a classic microscope it is possible to see that the surface of all bodies which look flat or smooth contains asperities and small holes. That motivated the research of contact with interpenetration describing the fact that the asperities may fill the holes. However, such an interpenetration must be strictly limited by a certain bound (denoted in the graphical abstract by the letter beta) to be in harmony with the mechanical reality.

This value is measured in every point of the surface of the deformable body on its normal (cf. the graphical abstract). To let it have a proper meaning, the contact boundary is assumed to be smooth enough in a certain sense. The value beta is assumed to be prescribed. Moreover, a constitutive relation p which assigns the normal stress to a given normal displacement is also assumed to be prescribed. This function is assumed to have a vertical asymptote at the point denoted by beta as it is depicted in the left picture of the graphical abstract.

Let us compare the methods:

1. The Signorini contact has 3 premises:
  A. at the contact the normal displacement must have sign because the motion through the object in contact is not possible;
  B. the normal stress has also sign because it must be a pressure;
  C. if there is no contact, there is no pressure (the mutual product equals zero everywhere on the contact part of a boundary or on a contact domain if e.g. plates are treated). The constitutive relation is in fact zero for values of non-contact, is multivalued with the image set equal the non-negative real half-axis in the point of contact and it is plus infinity elsewhere.
2. In the normal compliance approach the constitutive relation is finite on the whole real axis. It seems to be derived from the approximate problems to the Signorini contact as e.g. Yosida approximation.
3. In our model the constitutive relation is finite on the open interval ending at the point beta. Starting from it, it is in fact plus infinity. This is just depicted on the left part of the graphical abstract while its right part shows the whole body with the different parts of boundary, the zone of the interpenetration along its contact part and the foundation which need not be in an actual contact with the body – there can be a gap g between them. In such case there is a shift in the graph of p: g should replace 0 and beta + g should replace beta. The gap differs generally in different points of the contact boundary and is measured on the corresponding normal.

Let us remark that in the framework of the Signorini contact some kind of limited interpenetration can be also studied when the constitutive relation has a form of a non-decreasing function which is at some point multi-valued with a half-line being the image set. Such model describes a highly non-homogeneous body consisting of a completely penetrable cover and impenetrable core. It seems that such model has been studied in [6] for the first time. The model with limited interpenetration does not expect so strict inhomogeneity.

The co-published paper about the quasistatic contact [5] is the fifth in the row of papers about the existence of solutions for the contact problems with limited interpenetration. The first two covered the static contact – in [1] the coercive case and in [2] the semicoercive one have been studied. The further papers dealt with the dynamic frictionless contact for viscoelastic material. In [3] the boundary contact of a body and in [4] the domain contact of different models of plates have been analyzed.

The concept of contact with limited interpenetration I initiated and developed with an amazing and essential cooperation of C.Eck and after his very premature and tragic death partly with J. Stará in [1]. Unlike the Signorini contact, whose constitutive relation is multi-valued as described earlier (which leads to a well-known variational inequality defined on an appropriate cone as a weak formulation of the problem), the frictionless model with the limited interpenetration has a variational equation as its weak formulation. If the Coulomb friction is considered, the weak formulation remains a variational inequality.

The main method of solution of such kind of problems employs a suitable approximation which is here made by a pointwise non-decreasing system of non-decreasing non-negative functions having an affine growth at plus infinity approaching the constitutive relation. Such approximate problems are easily solvable because their “contact” term is nothing else than an additional well behaving Nemytskii operator.

The main task, the limit process to the original problem, is more difficult. Here I would like to concentrate on the problems with Coulomb friction in the framework of quasistatic problems. Let me describe briefly the methods leading to the proof of the existence of solutions published in Ref. [5].

When contact with Coulomb friction is treated, some information about some additional uniform regularity of approximate solutions, in particular about the uniform partial space regularity of the normal boundary stress on the support of the coefficient of friction (the closure of the set where it is non-zero) is vital and the same holds for the contact with limited interpenetration. Under some mild requirements on the given volume force and some stronger requirements on the smoothness of the contact part of the boundary it is proved that the boundary stress is a square integrable **function** there, which is crucial in that limit process.

In quasistatic problems the body is assumed to be deformed very slowly, hence the inertial forces in the dynamics are small and therefore neglected. Since the body is elastic, the velocity occurs in the friction term only. For such problems the time discretization (Rothe method) is natural in the process of solving it. As in the static problems the Euclidian norm in the Coulomb law is smoothened to get a variational equation. It is solved by a fixed-point approach for the finite system of time levels. Hence all results known for the static problem in [1] can be used and the space regularity result mentioned in the previous paragraph can be employed starting from here. The advantage is that the value of the previous time step which results from the velocity occurring only in the friction term is “pocketed” here in the gradient of the smoothing function which is constructed in such a way that the Euclidian norm of the gradient is everywhere bounded by 1. The fact that (unlike the static case) we have not an a priori estimate known ahead, implies that the first a priori estimate performed is formulated in the norms defined on the whole support of the coefficient of friction. Thus the bound of the admissible coefficient of friction is dependent on the form of the contact part of the boundary. After a standard transition to the nonsmooth problem (containing the original Euclidian norm) some information about time regularity is needed to allow the transition to the continuous time problem. Apart from the assumption (square integrability together with their generalized time derivatives) about the input data (the volume force, the boundary force on its free part and the given displacement on the fixed part of the boundary) this requires also a special bound on the multiplier norm of the coefficient of friction. To calculate this norm for different bodies may be difficult, usually only some estimates of it are available. Then the transition from the discrete to the continuous time is quite standard. The function and its time difference are prolonged to the time step intervals by the values in their left end. This allows us to get a limit which is a solution to the approximate problem and the mentioned regularity of the boundary stress remains valid with the bound for the norm which is independent of time.

The limit process to the original problem is based on the proved space regularity of the normal boundary stress as well as on its time regularity which in the quasistatic problems depends directly on the time regularity of the input data. This allows us to perform the limit processes, in particular in the friction term. The monotonicity of the approximate system helps here significantly. Some results from the original static paper [1] are employed here to prove that some weak limits, existing due to the reflexivity of the used spaces, are just those objects which are needed to be. Let us remark that this step, standard also when Signorini contact is treated, is omitted in the normal compliance approach for which our approximate solution is just a desired solution.

Finally the convergence of a selected subsequence of solutions to the problems with limited interpenetration to some solution of the corresponding Signorini problem is proved provided the depth of the interpenetration tends to zero. This depth does not influence the estimates of different norms to the solutions, because the normal stress was treated there as defined by the Green formula and not in the form of its expression by the constitutive relation. The proof, less straightforward than those made in papers [1], [2], [3], [4], may be seen as an alternative proof of the existence of solution to the quasistatic Signorini contact with friction. There are some differences with respect of Andersson's proof which is based on the appropriate Yosida approximations.

The common feature of refs. [1], [2], [3], [4], [5] is the proof of the existence of solution to problems posed. Of course, this has no straightforward impact to technical practice. However, it makes the usage of this model safe from the point of view of continuum mechanics. In particular, it was proved that the friction is in all cases dominated by the sum of the volume energy and the contact energy (defined by means of the integral of the function p), while those energies are dominated by external (the sum of volume and boundary) forces. Let us remark that for the appropriate domination of the friction energy the proof of the regularity of the boundary traction (as well as the boundary displacement) is really crucial, because it enables us to perform the required limit procedure. The results as well as the procedures performed in the proofs create a reliable basis for numerical analysts for their particular problems.

The question of uniqueness of solutions having a remarkable importance for numerical analysis remains mostly open. In Signorini static and quasistatic problems the uniqueness is proved for frictionless coercive problems and also the semicoercive problems are well mapped. It remains true for the corresponding problems in the model with limited interpenetration. However, just Coulomb friction brings a lot of uncertainty and, since the publishing the first existence results more than 40 years ago, the problem of uniqueness resists to all attempts to solve it. The dynamic Signorini contact has generically non-unique solutions and only some special additional requirements as e.g. energy conservation may lead to a choice of a unique solution having such properties. However, such results are extremely rare in the literature. Concerning the contact with limited interpenetration similar difficulties should be expected, although the situation may differ.

In practical applications the main task is an appropriate choice of the value beta and of the constitutive relation p. Some sensitivity analysis with respect to such choice is advisable. To preserve a good geometrical sense, beta should be smaller than the minimum (taken over the contact boundary!) of maximal radius of a sphere being inside the body in contact and containing a certain point of the contact boundary. In this case the normals from different points of the contact boundary cannot intersect in the interpenetration zone.

**I wish to announce that this approach is available for a vast amount of different practical contact problems as a reliable option to methods used up-to-now.** It seems that the framework of the contact with limited interpenetration is for this purpose physically better justified that the normal compliance approach.

## Declaration of Competing Interest

The author is not being aware of any possible conflict of interest related with the publication of this manuscript.
